# Preparation and Characterization of Microencapsulated Ethylenediamine with Epoxy Resin for Self-healing Composites

**DOI:** 10.1038/s41598-019-55268-7

**Published:** 2019-12-11

**Authors:** Liye Yuan, Tongqing Sun, Honglin Hu, Shuxia Yuan, Yu Yang, Rongguo Wang, Chunxiang Lyu, Fan Yang, Xiaoxuan Lyu

**Affiliations:** 1 Key Laboratory of Carbon Materials, Institute of Coal Chemistry, Chinese Academy of Sciences, Taiyuan, 030001 People’s Republic of China; 2 National Engineering Laboratory for Carbon Fiber Technology, Institute of Coal Chemistry, Chinese Academy of Sciences, Taiyuan, 030001 People’s Republic of China; 3 Center for Composite Materials and Structures, School of Astronautics, Harbin Institute of Technology, Harbin, 150080 People’s Republic of China; 4 Science and Technology on Advanced Functional Composites Laboratory, Aerospace Research Institute of Materials and Processing Technology, Beijing, 100076 People’s Republic of China

**Keywords:** Chemistry, Engineering, Materials science

## Abstract

Healing agent microcapsules have been used to realize self-healing for polymeric composites. In this work a novel kind of microcapsules encapsulating ethylenediamine (EDA) with epoxy resin as shell material were prepared by interfacial polymerization technology. The oil phase was epoxy resin prepolymer and carbon tetrachloride, and the water phase was EDA and deionized water. Under the action of emulsifier, a stable water-in-oil emulsion was formed. Then the emulsion was added to dimethyl silicone oil, stirred and dispersed, to prepare microcapsules. In addition, the factors affecting the preparation of microcapsules were studied. In this study, Fourier transform infrared(FTIR) was carried out to demonstrate the chemical structure of ethylenediamine microcapsules. Optical microscope(OM) and scanning electron microscope(SEM) were used to observe the morphology of microcapsules. Thermogravimetric analysis and differential scanning calorimetry were done to investigate the thermal properties of microcapsules. Permeability experiment and isothermal aging test were executed to verify the environment resistance of microcapsules. Results showed that EDA was successfully coated in epoxy resin and the microcapsule size was in the range of 50~630 μm. The synthesized microcapsules were thermally stable below 75 °C and perfect permeability resistance to ethanol solvent.

## Introduction

With the development of modern civilization, the application of composite materials is more and more extensive, and the technology is more and more mature. Nowadays we can see the composite materials in many fields, such as aeronautics and astronautics, automobile industry, electronic field, machinery industry, chemical, sports equipment, etc. Among them, resin-based composites are the most widely used. However, resin-based composites are easy to aging under the action of environment and loads, and crack occurs inevitably in the process of application for composites, and it is quite difficult to detect and maintain for engineers, when it is inside the composite materials. As time goes on, crack will continue to expand, and more crack will be developed, eventually the mechanical properties of the resin-based composites will be significantly reduced, leading to severe consequence. Therefore, early detection and healing of these injuries is very important for the application of resin-based composites.

At present, there is great interest in the research of intelligent materials with self-repairing function at home and abroad^1–4^, which is an emerging research technology. Researchers simulate the natural life process, so that the inanimate material also has the biological ability of automatic repair, it can automatically sense and respond to the stimulus. The self-healing process can be achieved via embedding a variety of containers^5^ such as capsules^6–11^, hollow fibers^12–14^ or vasculature systems^15–17^ into the matrix of composites. However, it is difficult to compound hollow fiber^11^ and vasculature systems^18^ with composites, and the weak ability to repair large crack with bond reconstruction^19^ limits their applications in self-repairing composite materials. Alternatively, microcapsule technology has been one of the most widely developed methods to fabricate self-healing materials, such as dicyclopentadiene^7,20,21^, amine^22^, and epoxy^23^ have been encapsulated in microcapsule successfully to apply for self-healing materials.

Nowadays, the field of microcapsule self-healing is developing quite rapidly. White *et al*.^7^ encapsulated liquid dicyclopentadiene (DCPD) via a urea-formaldehyde (UF) polycondensation. The DCPD microcapsules and 1st generation Grubby catalyst particles were embedded in an epoxy matrix, while the epoxy-based composites were fabricated. When the composites materials ruptures, these DCPD microcapsules break and outflow the liquid DCPD into the crack, where the dispersed Grubbs’ catalyst will be contacted, triggering ring-opening metathesis polymerization (ROMP) to form a cross-linked network, then the epoxy crack surfaces will be adhered back together. At last 10 wt% of endo-DCPD UF-microcapsules and 2.5 wt% of Grubbs’ catalyst were used to prepare an epoxy tapered double cantilever beam (TDCB) geometry, getting an average fracture toughness (K_IC_) recovery of 60% compared to the original epoxy after a healing time of 2 days. However, Grubbs’ catalyst is easily deactivated by amine curing agent in the composites, thereupon Rule *et al*.^24^ coated Grubbs’ catalyst with paraffin wax as shell materials of microcapsules to prohibit the catalyst from deactivation by the amine hardener and make it more easily dispersed in the epoxy matrix. To prepare microcapsules with appropriate strength and stability in surrounding medium, Yuan *et al*.^25^ introduced poly (melamine-formaldehyde) (PMF) as the shell material of microcapsule to encapsulate DCPD. Zhang *et al*.^6^ utilized etched glass bubbles as robust micro-containers to encapsulate primary amine and diisocyanate monomer. Fereidoon *et al*.^26^ prepared DCPD microcapsules with the urea-formaldehyde resin acting as shell materials which were modified by carbon nanotubes and aluminum oxide nanoparticles to improve the water resistance and thermal resistance of microcapsules. Yin *et al*.^27^ synthesized epoxy microcapsules with the urea-formaldehyde as the shell material, while latent hardener (CuBr2(2-MeIm4)) which is well soluble in epoxy was dispersed in epoxy composites during the manufacturing process of composites. When the crack generates and expands in the composites, the microcapsules break with the inside epoxy resin outflowing, and then react under the action of latent hardener (CuBr2(2-MeIm4)) to repair the crack.

However, there are several issues existed in these self-healing microcapsules systems, one is that the shell materials of microcapsules usually is different with the matrix of epoxy-based composites, so there is an interface problem between shell materials of microcapsules and composites, when the crack tip extends to the vicinity of microcapsule, it maybe expand along the interface between microcapsules and epoxy resin, making the microcapsule fail to fracture and lose the self-healing function. Another issue is that the reactivity of most healing agents is not high enough, either the reaction time is a little longer or reaction needs to be heated, which greatly affects the self-healing speed and effect. Anymore, catalysts, such as the Grubbs^7^ and Hoveyda-Grubbs^28^ catalyst, due to their high price and low versatility, they are difficult to be commercialized^29^. Alternative catalysts, such as DCPD and Toluene diisocyanate (TDI) has strong toxicity and is harmful to human body and environment. Based on the above three factors, in this study we selected epoxy resin as the wall material of the microcapsules to solve the interface problem between the microcapsules and the matrix of the composite materials, and the EDA which is more economical, lower toxicity and higher active as the core material to prepare the microcapsules.

EDA has low viscosity and good miscibility with epoxy resin. It can cure epoxy resin at room temperature, releasing heat which can further promote the curing speed. However, EDA as a kind of fatty amine curing agent of epoxy resin has properties of high volatility, low boiling point and good solubility, which is soluble in water and many sorts of organic solvent, so it is very difficult to be coated, that is why there have been rare reports of EDA being encapsulated successfully up to now. In this study, there are three key factors to synthesize the microcapsules successfully. The first one is that epoxy resin prepolymer is introduced to accelerate the polymerization reaction speed of epoxy resin to form compact shell material, prohibiting the EDA from volatilizing, and the second one is a small amount of deionized water is added to EDA to form complex, and stable emulsion of water-in-oil is prepared successfully. The third one is high viscosity dimethyl silicone oil is used as dispersion medium. Because neither epoxy resin nor water is soluble in dimethyl silicone oil, stable tiny drops of emulsion can form when the emulsion is added in under agitation. Anyway, high viscosity of dimethylsiloxane allows tiny droplets of emulsion to disperse very steadily without the existence of emulsifiers. Then the EDA microcapsules are synthesized successfully finally.

## Experimental

### Materials

EDA and dimethyl silicone oil were purchased from Tianjin Fengchuan Chemical Reagent Technology Co., Ltd, China. Epoxy resin E51, used as the shell material of microcapsule, were purchased from Bluestar Wuxi Resin Factory. Carbon tetrachloride, used as organic solvent, was supplied by Tianjin Fuyu Fine Chemical Co., Ltd. Span-80 and Tween80 used as emulsifiers were obtained from Tianjin Guangfu Fine Chemical Research Institute.

### Preparation of microcapsules

The microcapsules containing EDA were synthesized via interfacial polymerization in a water-in -oil emulsion. Firstly, the oil phase, 5 g epoxy resin E51 and 0.65 g EDA, was prepared by mixing and put into electric blast drying oven keeping the temperature at 50 °C for 10 min, then measurements of 1 g carbon tetrachloride and 3 wt% emulsifier were added into the epoxy resin, and they were stirred to form homogenous phase. Secondly, the water phase, 1 g EDA and 0.1 g deionized water were mixed together to form solution. Thirdly, the water phase was added into the oil phase dropwise to form stable emulsion by magnetic stirring for 20 min. Fourthly, the emulsion was added to 80 ml dimethyl silicone oil which was used as a continuous phase in a 250 ml glass beaker and the mechanical stirring speed was adjusted to 200 r/min. After that, the system further reacted for another 24 h. the obtained suspension of EDA microcapsules was filtered and rinsed with carbon tetrachloride and ethanol successively. Finally, the collected EDA microcapsules were air-dried at room temperature for 8 h.

In order to explore the optimal synthesis process of EDA microcapsules, orthogonal experiments were designed by selecting four influencing factors: raw material ratio, stirring speed, type of emulsifier and reaction time. Table 1 shows the main test levels and process parameters of orthogonal test.

**Table 1:** Main Process Parameters and Test Levels of Orthogonal Test.

Level	Weight ratio of EDA/EP A	Agitation rate(rpm) B	Reaction time(h) C	Emulsifier D
1	1:5	100	2	Span80
2	1.5:5	200	3	Span80, Tween80
3	2:5	300	4	—

### Characterization

The EDA microcapsule morphology were observed by using an Scanning electron microscope (SEM, QUANTA200) and optical photo microscope (OM, BX51). The chemical structure of the EDA microcapsules was analyzed by FTIR spectrometer (AVATAR 370). The FTIR spectra of microcapsule samples were compared with the spectra of shell materials, i.e. epoxy resin cured with EDA and the spectra of core materials, i.e. EDA with a small account of water. The activity analysis of core material was investigated by using DSC at a heating rate of 10 °C/min in a nitrogen atmosphere. 10 mg of mixture of EDA microcapsules and epoxy resin was weighed according to the weight ratio of 7:5 to heat from room temperature to 250 °C, comparing with 10 mg mixture of epoxy resin and EDA with the ratio of 10:1. TGA (PYRIS 6) was used to investigate thermal property of microcapsules at a heating rate of 10 °C/min. Though weighting the microcapsules several times after treatment at 50 °C and 80 °C respectively in an electric blast drying oven, the isothermal aging experiment was carried out. The permeability experiments were performed according to the following method of weighing the microcapsules which were immersed in anhydrous ethanol for different time after drying at 50 °C.

## Results and Discussion

### Microencapsulation process

**Figure 1:**
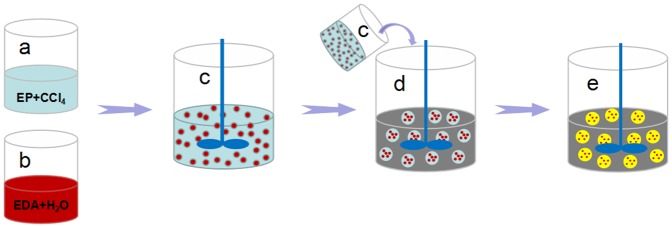
Synthesis diagram of EDA microcapsules.

EDA microcapsules are synthesized by interfacial polymerization. Figure 1 shows the synthesis diagram of EDA microcapsules. Because the epoxy resin is oil-soluble and the ethylenediamine is water-soluble, the microcapsules are synthesized by water-in-oil system. Firstly, epoxy resin prepolymer is prepared by mixing epoxy resin and EDA at a mass ratio of 100:13 to ensure enough -NH_2_ supplied without consuming the core material. Then epoxy resin prepolymer, carbon tetrachloride and emulsifier are mixed to form a continuous phase [Fig. 1(a)], while EDA and deionized water are blended as the dispersed phase [Fig. 1(b)]. Secondly, the dispersed phase is added to the continuous phase to form a stable emulsion by mixing [Fig. 1(c)]. Thirdly, the emulsion is added to dimethyl silicone oil, and the whole system is stirred all the time [Fig. 1(d)]. After the epoxy resin prepolymer is cured to be rigid polymer shell, the EDA as core materials was coated to form microcapsule [Fig. 1(e)].

### Influencing factors of preparation of the EDA microcapsules

As discussed above, the EDA microcapsules are fabricated via interfacial polymerization of epoxy resin with EDA. Therefore, the preparing condition such as weight ratio of EDA/EP prepolymer, agitation rate, the reaction time and emulsifier would influence the preparation of microcapsules. In this section, the influence factors of preparation of the EDA microcapsules were investigated. For this purpose, orthographic factorial design was applied. Table 2 show the results and analysis of the effects of the four factors at three levels (refer to Table 1).

**Table 2:** Orthogonal experiments.

Synthetic parameters				Characterization results			
A	B	C	D	Encapsulation results	Situation of microcapsules	Size distribution (μm)	Mean size (μm)
1	1	1	1	Failure	—	—	—
1	2	31	2	Good	Sphere or ellipsoid, no adhesion	50~630	200
1	2		2	Failure	—	—	—
1	3	3	2	Good	Perfect sphere, no adhesion	100~400	300
2	2	3	1	Failure	—	—	—
2	3	2	2	General	Slight adhesion	80~350	280
3	1	3	2	Failure	Serious adhesion	250~1000	600
3	2	2	1	Failure	—	—	—
3	3	2	2	Failure	Serious adhesion	110~480	330

According to Table 2, the second and the fourth groups were found to have the best encapsulation effect. The shell material is dense, the appearance is crystal clear, the mechanical properties are perfect.

### Effect of core-shell ratio on the preparation of microcapsules

**Figure 2:**
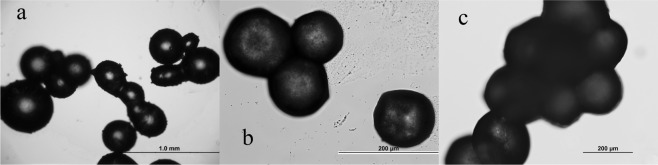
The OM images of EDA microcapsules at different mass core-shell ratios (a = 1:5, b = 1.5:5, c = 2:5).

The coating rate of microcapsules is directly affected by the core-shell ratio. Figure 2 shows OM photos of microcapsules synthesized at three different core-shell ratios (1:5, 1.5:5, 2:5). It can be seen from the photos that when the core-shell ratio is 1:5, the coating result is the best, and the surface of the microcapsules is smooth and compact [Fig. 2(a)]. When the core-shell ratio increased to 1.5:5, the mechanical strength of the shell material of microcapsules decreases, and the shell material shows slight adhesion [Fig. 2(b)]. When the ratio increases to 2:5, the mechanical strength of the shell material is very low, and the microcapsules prepared are easy to break, and the adhesion of microcapsules is very serious [Fig. 2(c)]. Therefore, the optimal core-shell ratio is 1:5.

### Effect of stirring rate on the preparation of microcapsules

**Figure 3:**
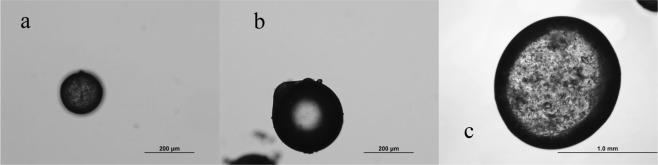
The OM images of EDA microcapsules at different stirring speed (**a** 250 r/min, **b** 200 r/min, **c** 100 r/min).

Figure 3 shows the prepared EDA microcapsules under different stirring rate. The size of EDA microcapsules decreases with the increase of stirring rate, and the size distribution range also narrows. In order to obtain the smaller particle size and narrow distribution of EDA microcapsules, stirring speed can be controlled in 200~250 r/min.

### Effect of reaction time on mechanical strength of microcapsules

**Figure 4:**
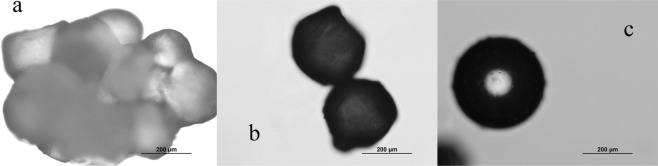
The OM images of EDA microcapsules after different reaction times (**a** 12 h, **b** 18 h, **c** 24 h).

Different reaction times will lead to different curing degrees of the wall materials. As shown in Fig. 4, the optical photos of EDA microcapsules obtained after 12 h, 18 h and 24 h reaction time respectively. When the reaction time lasts for 12 h, the curing degree of the shell material of microcapsules is not enough, and the adhesion of microcapsules is very serious after filtration and washing [Fig. 4(a)]. The curing degree increases when the reaction time adds to 18 h, while the mechanical strength of the shell material is greatly improved, and the adhesion between microcapsules is greatly improved after filtration and washing [Fig. 4(b)]. When the reaction time lasts 24 h, microcapsules without adhesion are obtained. The shape of microcapsules is spherical, and the mechanical strength of the shell material is very good [Fig. 4(c)]. According to the effect of reaction time on mechanical strength of the microcapsules shell material and adhesion between microcapsules, the reaction time should be controlled to about 24 h.

### Effect of emulsifier on the preparation of EDA microcapsules

**Figure 5:**
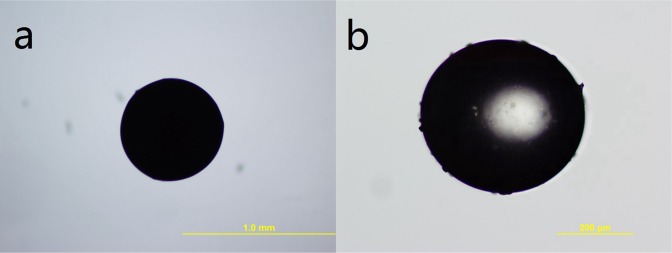
The OM images of microcapsules prepared by different emulsifiers (**a** Span80, **b** Span80:Tween80 = 1:1).

Two kinds of emulsifiers are chosen in this study, non-ionic emulsifier Span80 and compound emulsifier composed of Span80 and Tween80. The mass ratio of Span80 to Tween80 is 1:1 in the compound emulsifier. Figure 5 shows OM photos of EDA microcapsules prepared with Span80 and compound emulsifier composed of Span80 and Tween80, respectively. Epoxy microspheres [Fig. 5(a)] rather than EDA microcapsules can be obtained with single Span80 acts as emulsifier, because formed emulsion is unstable. When the emulsion is added to the dimethyl silicone, EDA can not be well coated in the epoxy resin. With the extension of reaction time, before the epoxy resin cures, EDA has vaporized into the air to form epoxy microspheres. While stable emulsion can be formed with compound emulsifier composed of Span80 and Tween 80, and EDA microcapsules are prepared successfully at last [Fig. 5(b)].

### Chemical structure of microcapsules

**Figure 6:**
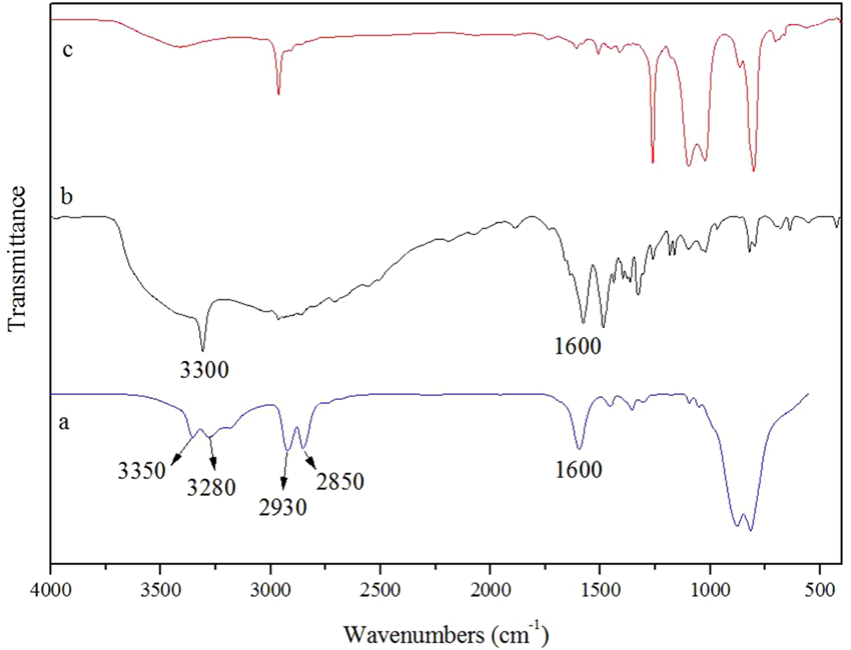
FTIR spectra of (**a**) core material, (**b**) microcapsules, (**c**) shell material.

Figure 6 shows the FTIR spectra of core material EDA and deionized water, microcapsules, epoxy resin shell material. From the infrared spectra of the core material, the absorption peak at 3350 cm^−1^ and 3280 cm^−1^ represent the symmetric stretching vibration of -NH_2_, while the absorption peak near 1600 cm^−1^ is the shear vibration of -NH_2_ in EDA. The absorption peak near 2850 cm^−1^ and 2930 cm^−1^ represent the stretching vibration of -CH in EDA. However, there is no absorption peak at 3350 cm^−1^, 3280 cm^−1^ and 1600 cm^−1^ in the infrared spectra of the shell material, indicating that there is no -NH_2_ in the shell material, and all the -NH_2_ in EDA reacts with the epoxy group. Moreover, the existence of the absorption peak near 3300 cm^−1^ and 1600 cm^−1^ in the infrared spectra of EDA microcapsules, which represents the stretching vibration and shear vibration of -NH_2_ respectively, indicates the presence of -NH_2_ in the microcapsules and further proves that EDA is coated in microcapsule successfully.

### Microcapsule surface morphology and size distribution

**Figure 7:**
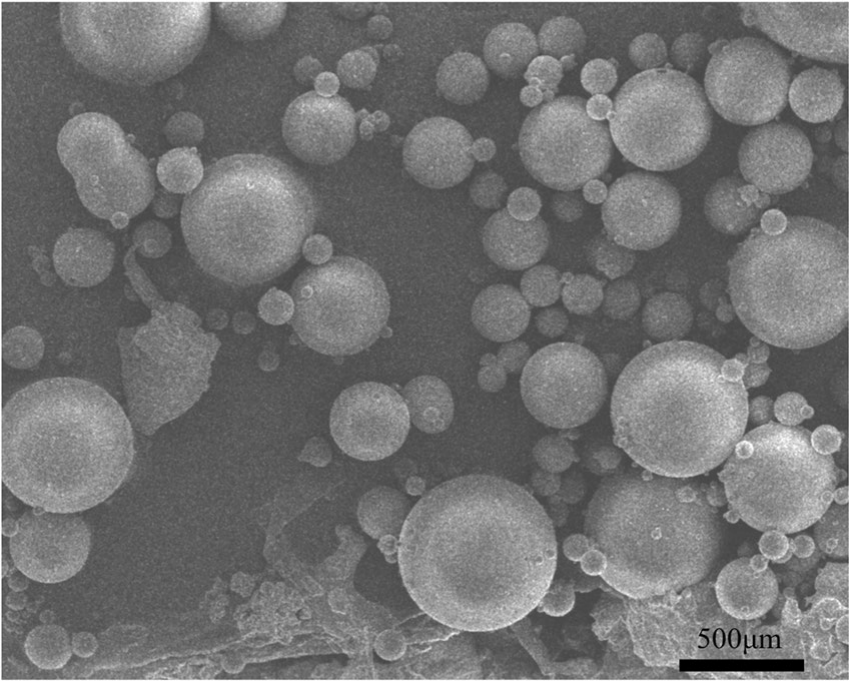
The SEM micrograph of EDA microcapsules.

**Figure 8:**
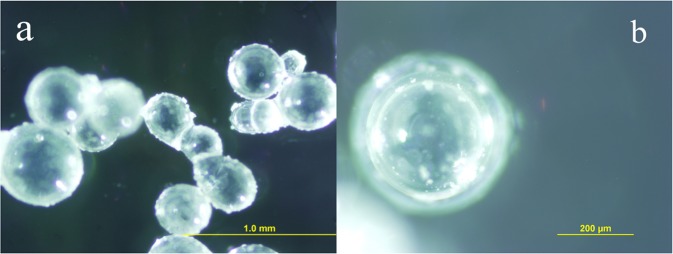
OM micrographs of prepared EDA microcapsules.

**Figure 9:**
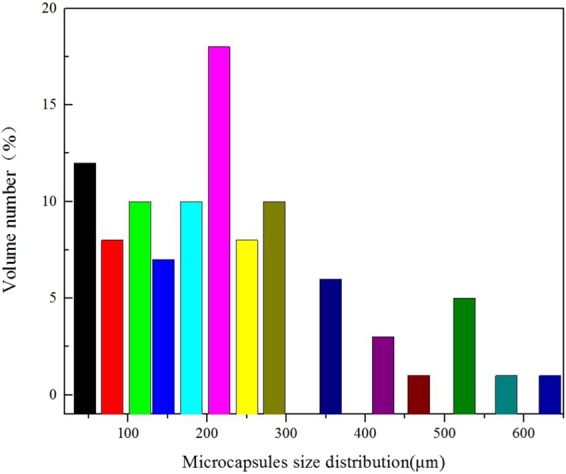
Size distribution of EDA microcapsules.

Figure 7 shows the SEM micrographs of EDA microcapsules sample. The outside surface of EDA microcapsules is smooth. The particle size distribution of microcapsules was analyzed by scanning electron micrograph. The average particle size of microcapsules was calculated by the size of 100 random microcapsules. Figure 8 shows the OM micrographs of EDA microcapsules sample. According to optical reflection theory, when light travels to different substances, it will reflect on the interface. So it is obvious that there are many EDA droplets inside the microcapsules from the OM micrographs, which proves that the EDA is coated in microcapsules successfully. Figure 9 shows the size distribution of the EDA microcapsules is between 50~630 μm, and the mean diameter is 200 μm. The uneven size distribution is due to the fact that the liquid flows rapidly around the agitator in the stirring process, so the microcapsules far away from the agitator have larger size, while the microcapsules close to the agitator have smaller size. The average particle size of the microcapsules can be controlled by adjusting the stirring speed of agitator.

### Thermal stability of microcapsules

**Figure 10:**
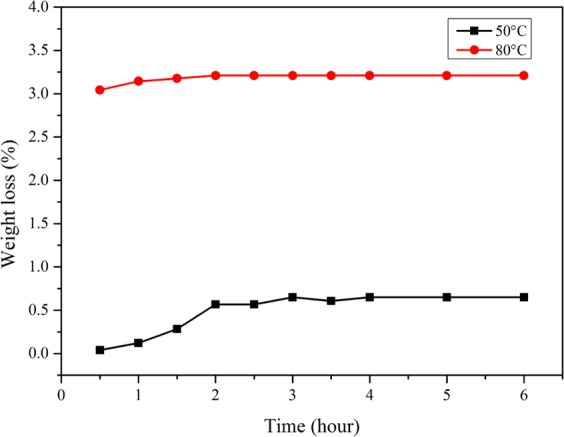
Weight loss of microcapsules at different time under the condition of 50 °C and 80 °C.

The isothermal aging experiments of EDA microcapsules were carried out. Figure 10 shows the curve of weight loss of ethylenediamine microcapsules at periodic time intervals at 50 °C and 80 °C, respectively. Under the condition of 50 °C for 0.5 h, the weight loss is about 0.04%. With the extension of exposure time, the weight loss of the microcapsule increased slowly. After about 2 h, the weight loss increases to about 0.567%, and then the mass of the microcapsule remains almost unchanged. It proves that the microcapsule has excellent thermal stability at 50 °C. The weight loss in the first two hours is mainly caused by the volatilization of ethylenediamine molecules near the outer layer of microcapsules along the micropores. In addition, under the condition of 80 °C, the weight loss of microcapsules has the same evolution law with the situation of 50 °C. For the first 0.5 h the weight loss is about 3.04%, and then the weight loss increases rather slowly, and after about 2 h, the mass of microcapsules keeps nearly unchanged. It indicates that when the temperature is lower, the ethylenediamine closest to the outer layer of microcapsules will volatilize and permeate firstly. With the increase of temperature, the permeation and volatilization of ethylenediamine extend to the interior of the microcapsules, so there are more core materials would escape from the microcapsules. Anyway, the permeation and volatilization of microcapsules core material is more sensitive to temperature than time. Once the surrounding temperature of microcapsules reaches a certain value, corresponding to depth of ethylenediamine will escape from the microcapsules. This process lasts about 2 h, even if the heat preservation time is lengthened, the weight of microcapsules will no longer change, which indicates that the synthesized microcapsules has excellent thermal stability.

**Figure 11:**
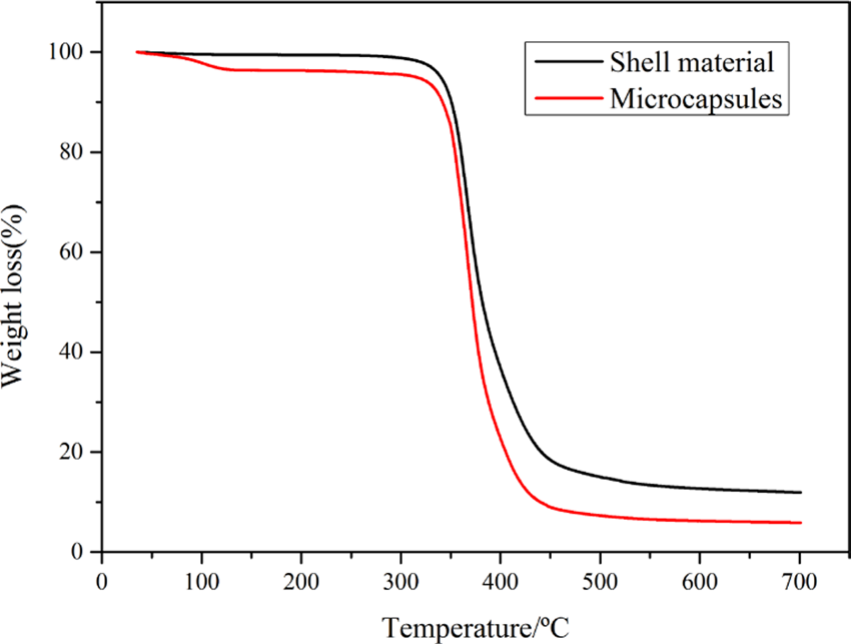
TGA curves of EDA microcapsules and epoxy resin shell materials.

Figure 11 shows the TGA curves of microcapsules and shell materials. The curve of shell materials indicates that the weight loss at 300–450 °C is mainly due to the decomposition of epoxy resin. The curve of microcapsules is consisted of two stages. In the first stage from 50 to 120 °C, the ethylenediamine escapes from the exterior layer to interior layer of microcapsules. With the increase of temperature, more and more EDA volatilize from the internal microcapsules, until approximate 120 °C the weight of microcapsules nearly no longer varies, which indicates that all the EDA is gone. The second stage is the same with shell materials from 300–450 °C, mainly being due to the decomposition of epoxy resin. As a consequence, the TGA curve proves that EDA is coated inside microcapsules successfully, and the core content is about 5%. The thermal resistance of EDA microcapsules is under about 120 °C.

**Figure 12:**
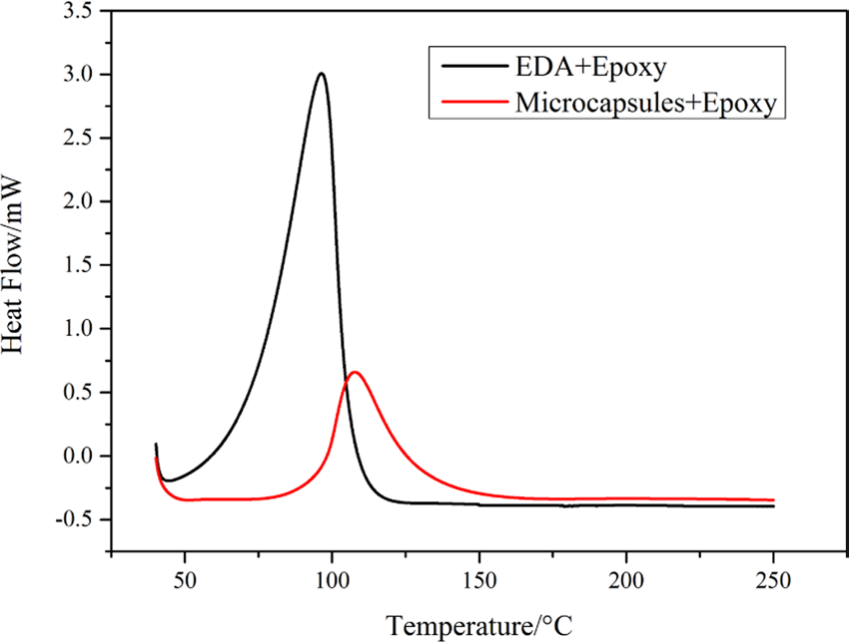
DSC curves of mixture of ethylenediamine and epoxy resin, microcapsules and epoxy resin respectively.

In order to verify whether ethylenediamine retains reactivity after it is coated in epoxy resin, DSC tests are performed on mixture of ethylenediamine and epoxy resin, microcapsules and epoxy resin respectively. The experimental results are shown in Fig. 12. An obvious exothermic reaction is detected in the heating up process of the mixture of EDA and epoxy resin (black curve, the peak temperature is 96.4 °C). Similarly, an apparent exothermic peak is detected substituting microcapsules for EDA in the heating up process (red curve, the peak temperature is 109 °C). However, the exothermic reaction peaks are not at the same position that is due to the protection of the epoxy resin shell materials, which prohibits the contact of EDA with uncured epoxy resin. With the increase of temperature, the EDA start to permeate and volatilize from the interior microcapsules and react with the uncured epoxy resin. Therefore, a higher temperature exothermic peak is detected at this time. The result of DSC curves indicates that the EDA is encapsulated in epoxy resin successfully and the encapsulated core material has excellent reactivity. In addition, from the red curve of Fig. 12, the reaction peak begins at about 75 °C, which proves that the thermal resistance of synthesized EDA microcapsules is rather perfect below 75 °C.

### Permeability of microcapsules

**Figure 13:**
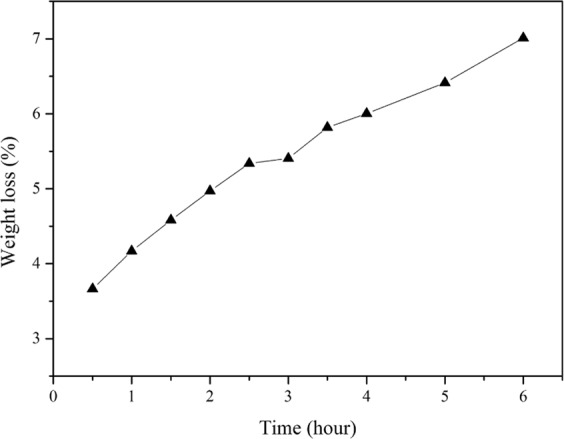
Permeability test curve of EDA microcapsules.

In order to analyze the compactness and sustained-release of microcapsule wall materials, the permeability experiment of EDA microcapsules was analyzed (Fig. 13). The result shows that the weight loss of microcapsules increases gradually with the extension of soaking time in ethanol. The weight loss of microcapsules increases from 3.66 wt% to 7.01 wt% after soaking in ethanol for six hours, which indicates that ethanol causes swelling in epoxy resin and diffuses into the microcapsules, making the EDA permeate from the microcapsules. Consequently, there is a certain level permeability to ethanol for the prepared EDA microcapsules.

## Conclusions

Epoxy resin as shell material and EDA as core material, the microcapsules are prepared successfully at last. This is achieved by forming a stable emulsion of epoxy resin and EDA, and occurring interfacial polymerization of epoxy resin to coat the core material and forming microcapsules with the dispersion function of silicone oil. Then the effects of core-shell ratio, stirring rate, reaction time and emulsifier on the preparation of EDA microcapsules are researched in this study. The optimum synthesis parameters are obtained. The best ratio of core-shell material is 1:5, and when the stirring rate is 250 r/min, microcapsules diameter is in a wide range of 50~630 μm and the mean diameter is about 200 μm. The optimal reaction time is 24 h. the results to prepare EDA microcapsules of compound emulsifier composed of Span80 and Tween80 is better than single Span80 emulsifier. The TGA curves prove that EDA is coated inside microcapsules successfully, and the core material content is about 5%. The DSC tests prove that the EDA in microcapsules has reactive activity and EDA microcapsules can have a good heating resistance below 75 °C. Anyway, there is a certain level permeability to ethanol for EDA microcapsules. In general, this work provides a novel preparation method of fatty amine curing agent microcapsules.
